# Pharmaceutical drugs supporting regeneration of small-intestinal mucosa severely damaged by ionizing radiation in mice

**DOI:** 10.1093/jrr/rrt077

**Published:** 2013-05-31

**Authors:** Hiroshi Ishihara, Izumi Tanaka, Haruko Yakumaru, Mika Tanaka, Kazuko Yokochi, Makoto Akashi

**Affiliations:** Research Program for Radiation Medicine, Research Center for Radiation Emergency Medicine, National Institute of Radiological Sciences, 4-9-1, Anagawa, Inage-ku, Chiba-shi, Chiba 263-8555, Japan

**Keywords:** radiation accident, thyroid hormone, histamine, glucagon, anabolic steroid

## Abstract

Accidental exposure of the abdomen to high-dose radiation leads to severe consequences initiated by disruption of the mucosa in the small intestine. Therapeutic options are limited, even though various treatments have been investigated, particularly in the field of regenerative therapy. In order to identify readily available treatment methods, we included several current pharmaceutical drugs, for which the clinical trials have already been completed, in tests on mice that had undergone severe mucosal damage by radiation. The drugs were injected into mice 24 h after exposure to 15.7 Gy X-rays. The effects of the drugs on the damaged mucosa of the small intestine were evaluated using early regeneration indices [the expression of *c-myb* mRNA, and proliferation of epithelial cells in the form of microcolonies (MCs) by Days 4 and 5 post-irradiation] and the survival rate of the mice. Enhancement of mucosal regeneration at Day 4 (*c-myb*: *P* < 0.01, MC: *P* < 0.05) and improvement of the survival rate (*P* < 0.05) were observed when a clinical dose of gonadotropin, a stimulator of androgen, was injected. Similarly, a clinical dose of thiamazole (which prevents secretion of thyroid hormone) stimulated mucosal growth by Day 5 (*c-myb*: *P* < 0.01, MC: *P* < 0.05) and also improved the survival rate (*P* < 0.05). The nonclinical drugs histamine and high-dose octreotide (a growth hormone antagonist) also gave significant survival-enhancing benefits (*P* < 0.01 and *P* < 0.05, respectively). These results can be used to construct therapeutic programs and applied in various experimental studies to control the regeneration of damaged mucosa.

## INTRODUCTION

Accidental high-dose radiation exposure causes a range of levels of damage in multiple organs [1]. Although hematopoietic damage can be restored by cytokines [2], failure in the intestinal mucosa leading to uncontrollable leakage of fluid is critical [3]. Repair of the mucosa by pleiotropic cytokines and grafted mesenchymal stem cells may be established in the future [4], but in the interim research is necessary for the development of methodology to regenerate damaged mucosa. Pharmaceutical drugs for which the safety testing has been completed are the usual choice in hospitals. Although a number of studies on radioprotectors—effective when injected before irradiation—have been reported, studies on drugs that ameliorate radiation damage when introduced after exposure are limited [5]. As an example of the latter, the peptide hormone family of glucagon and glucagon-like peptide-2, are known to stimulate proliferation of intestinal mucosa and regeneration of damaged mucosa [6–8]. They are available as medical drugs and used clinically in treatments for Crohn's disease.

We previously reported on the effects of steroid hormones in the mouse, and showed that regeneration of damaged mucosa is stimulated by clinical doses of anabolic steroid, but inhibited by high doses of estradiol [9]. These findings indicate that mucosal damage from radiation can be alleviated by anabolic steroid administration, but may be aggravated by follicle hormones administered for other purposes. This data on the effects of pharmaceutical drugs on the repair of mucosal damage are valuable in the choice of drugs for patients with severe accidental irradiation. In this study, we aimed to identify candidate therapeutic drugs effective against intestinal radiation damage, and to obtain data for further studies on mucosal regeneration.

Based on the pharmacological mechanism of action, we focused on gonadal hormones, thyroid hormones and growth hormone systems, since they have potential to affect mucosal regeneration. Pharmaceutical drugs that are known to modify these systems were injected into mice previously exposed to high-dose radiation, and the effects on the early regeneration of the mucosa and the survival rate were compared. Additionally, as an example of a locally acting hormone without direct effects on cell growth, we examined histamine, which is known to be a biogenic amine able to protect intestinal mucosa and epithelial cells [10, 11].

## MATERIALS AND METHODS

### Mice, irradiation and drugs

Male C3H/He inbred mice of 7 weeks of age (obtained from the Japan SLC Co.) were acclimatized at five per cage for 1 week in an animal room maintained at 23°C and 55% humidity, lit from 07:00 to 19:00 (‘Daytime 00:00 to 12:00’) with white light at 10–50 lx. Mice were treated in accordance with the Guidelines for Proper Conduct of Animal Experiments (Science Council of Japan). For homogenous X-irradiation of mice, up to ten mice were set in a cylindrical irradiation container with rotation as described previously [12]. From Daytime 02:00 to 05:00, the container was placed in the concentric irradiation field of an X-ray generator (PANTAK HF-320, Shimadzu Corp., Japan), and the mice were irradiated at 15.7 Gy at a focus-sample distance of 700 mm with a dose rate of air-kerma of 0.50 ± 0.05 Gy/min. The radiation doses were monitored by field measurements by the Department of Technical Support and Development of our Institute.

For survival studies, 15–50 mice from at least three different batches were used for the treatment groups. Mice were anesthetized by pentobarbital (80 mg/kg body-weight) and the abdomens of the mice, including the whole stomach and intestine, were X-irradiated at 15.7 Gy with shielding of the upper thorax by 3-mm tungsten sheet. Nutrients and drugs were injected from Day 1–7 after the exposure.

### Pharmaceutical drugs and doses

Doses of drugs were determined by recommended daily doses for patient in Japan with modifications (see Results). From 24 h after irradiation, therapeutic drugs were injected daily, at Daytime 02:00, combined with nutrients (FulCaliq^®^, Mitsubishi Tanabe Pharma Corp., Japan) at 20 ml/kg body-weight, and broad spectrum antibiotic [ceftazidime (LKT Labs, Inc.)] at 80 mg/kg body-weight (corresponding to the maximal daily dose for treatment of infection). Drugs used in this study were as follows: 100 units/kg body-weight (corresponding to the maximal daily dose for sterilitas virilis therapy) of human chorionic gonadotropin (from urine of pregnant woman, Wako Pure Chem. Ind. Ltd, Japan); 1 mg/kg (corresponding to an emergency dose for hyperthyroidism) of thiamazole (2-mercapto-1-methylimidazol, Wako); 1 mg/kg (125 times daily maximal dose for hypothyroidism) of thyroxine sodium 5aq. (Wako); 1 mg/kg (twice standard daily dose) of octreotide acetate (long-acting type, Sandostatin LAR^®^, Novartis Pharma); 0.1 mg/kg (five times standard daily dose) of recombinant rat growth hormone (rrGH, Wako); 40 mg/kg (20% of lowest published toxic murine dose as per RTECS) histamine dihydrochloride (Wako); 30 mg/kg (10 times standard daily dose) of diphenhydramine hydrochloride (Wako); and 0.2 mg/kg (10 times dose for emergency treatment for hypoglycemia) of human glucagon hydrochloride (Wako). Nandrolone with prolonged action (19-nortestosterone decanoate, Deca-Durabolin^®^, Japan Organon) was injected once at Day 1 with soybean oil at 50 µmole/kg (20 times standard therapeutic dose) into the inguinal region [9].

### Histochemical studies

As an histological indicator of mucosal regeneration of the small intestine, microcolonies [13] with BrdU-incorporated cells as seen in vertical section were used [9]. Two hours before killing, mice were subcutaneously injected with BrdU/FdU (10:1) at 30 mg/kg body-weight. Full-length small intestines were isolated from mice at Daytime 04:00 to 05:00 and were immersed in lactate Ringer's solution (Lactec^®^G, Otsuka Pharmaceutical Factory Inc., Japan) containing 10 μg/ml of papaverine hydrochloride (Dainippon Sumitomo Pharma Co., Ltd, Japan) and scopolamine butylbromide (buscoban^®^, Nippon Boehringer Ingelheim Co., Ltd, Japan) at 4°C. Using a standard method, all 15 mm-length segments of the intestine were fixed with formaldehyde in parallel, embedded with paraffin, sectioned, and the BrdU-incorporated cells were stained using an Amersham Cell Proliferation kit (GE Healthcare UK, Ltd); nuclei were stained using hematoxylin. The total numbers of microcolonies containing >10 or >30 BrdU-positive cells at Day 4 or Day 5, respectively, per vertical intestine mm-length were calculated.

### C-myb mRNA quantification by real-time RT-PCR

As a molecular indicator of crypt regeneration, we measured mucosal levels of mRNA for *c-myb* protooncogene expressed in early stem cells [14]. Exact quantification of mRNA rates of c-myb/GAPDH by real-time RT-PCR has been described elsewhere [9, 15–17]. In brief, the total RNA from the mucosa of the terminal of the ileum (in the length of 30 mm of mice sacrificed at a time between Daytime 04:00 and 05:00) was prepared using the RNeasy Mini Kit^®^ (Qiagen GmbH, Germany). Template cDNAs were synthesized using AMV reverse transcriptase (Takara Bio Inc., Japan) at 1 unit/300 ng of total RNA, as measured by electrophoretic image scanning. Using SYBR^®^ Green PCR Master Mix, MicroAmp^®^ Fast 96-Well Reaction Plates, and ABI Prism7500Fast^®^ Sequence Detection Systems with 96 wells (Applied Biosystems Inc., CA, USA), quantitative real-time RT-PCR in quadruplicate of the template cDNAs corresponding to 10–40 ng of total RNA/well was performed in the presence of internal standard DNA at 20–160 zepto mole/well. Primer sets were as follows: GAPDH set (fw = 5′-TGGCCAAGGTCATCCATGACAAC-3′, rv = 5′-TCCAGAGGGGCCATCCACAGTCTTCTG-3′), *c-myb* set (fw = 5′-TGACACCTGTATCAGAAGATGAAGACA-3′, rv = 5′-AGGCACCACTGCATGGCT-3′).

### Statistics

Drug effects on mucosal regeneration were quantified by multiple comparisons to estimate the *P* value by Dunnett Test following one-way ANOVA of the data for the rate of BrdU-incorporated microcolony growth and the RNA ratio of *c-myb*/GAPDH. The Newman–Keuls Test was also used. To determine drug effect on the survival of abdominally irradiated mice, a log-rank test following the Kaplan–Meier method was performed and the significance was determined.

## RESULTS

### Quantitative indicators to estimate regeneration of damaged mucosa

**Fig. 1.**
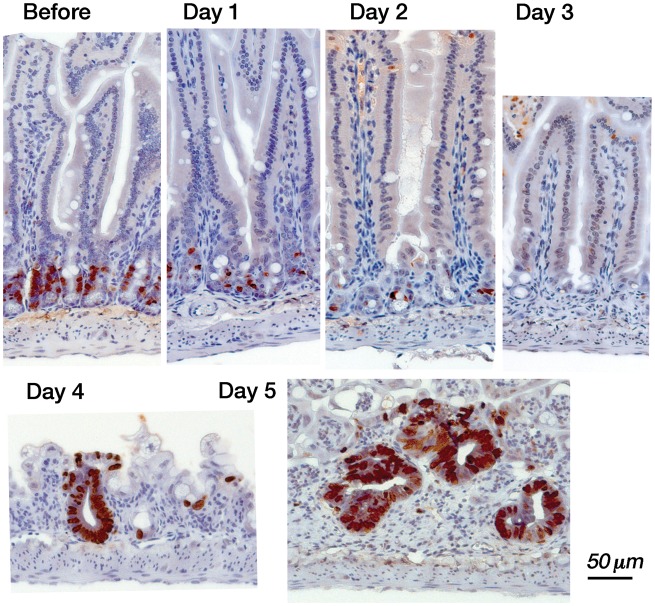
Damage and regeneration of the mucosa of the small intestine after a lethal dose of radiation. Proliferating mucosal epithelial cells with BrdU-incorporated nuclei (brown) were located in the middle of the crypts (before). After X-irradiation at 15.7 Gy, the proliferating cells in the crypts decreased each day (Day 1, Day 2), then disappeared (Day 3). Regeneration of the epithelium (as BrdU-positive microcolonies) was observed at Day 4. The size and the number of the microcolonies increased by Day 5.

Mucosal damage and the regeneration process in the small intestine can be observed after whole-body irradiation of mice at 15.7 Gy [9]. As shown in Fig. 1, the numbers of BrdU-positive proliferating cells in the crypts decreased, reaching a minimum on Day 3 after the exposure. Subsequently, microcolonies containing BrdU-positive cells were formed by Day 4, and increased by Day 5 as part of the early regeneration process of the mucosal epithelium. Finally, all the whole-body irradiated mice died between Day 5 and 8. When the hematopoietic cells were protected by local shielding of the costal marrow mice, regeneration of the epithelial area expanded to construct villi while repairing the nutrient absorption after Day 8, and half of the mice survived [9]. Since the early regeneration process occurring on Days 4 and 5 strongly affects the recovery of the damaged intestinal mucosa, we used two quantitative indicators to examine the effects of the drugs. First, the mucosal mRNA rate for *c-myb*/GAPDH (Table 1), which is expected to indicate the early activation of the cryptic stem cells towards the differentiated crypt [14, 18], was examined. As another regeneration indicator, we obtained the ratios of the numbers of BrdU-positive microcolonies, reflecting the proliferating cells, per mm-mucosal length in vertical section for the whole small intestine (Table 2).

**Table 1.** Messenger RNA rates of c-myb/GAPDH (×10^−3^) in mucosa of ileum of mice whole-body-irradiated with 15.7 Gy X-ray

Drugs	Day 4	Day 5
**Saline**	0.78 ± 0.15	1.62 ± 0.26
**Nandrolone**	1.69 ± 0.36	4.95 ± 1.35*
**Gonadotropin**	3.08 ± 0.92*	4.81 ± 0.28**
**Glucagon**	0.72 ± 0.07	3.56 ± 0.93
**Thyroxine**	1.58 ± 0.68	4.10 ± 1.41
**Thiamazole**	1.65 ± 0.33	5.85 ± 1.21*
**Growth hormone**	1.08 ± 0.38	2.96 ± 0.82
**Octreotide**	1.66 ± 0.68	2.63 ± 0.92
**Histamine**	1.66 ± 0.30	1.78 ± 0.34
**Diphenhydramine**	2.68 ± 0.30*	2.81 ± 0.69

Averages and the standard error among 3–4 mice are indicated. *P < 0.01, **P < 0.05 by one-way ANOVA using Dunnett Test for multiple comparisons with saline treatment as a control.

**Table 2.** Ratio of the number of BrdU-positive microcolonies per mucosal length (mm) of a vertical section of the small intestine

Drugs	Day 4	Day 5
**Saline**	0.130 ± 0.006	0.264 ± 0.016
**Nandrolone**	0.132 ± 0.006	0.497 ± 0.030*
**Gonadotropin**	0.217 ± 0.037**	0.301 ± 0.058
**Glucagon**	0.071 ± 0.005†	0.436 ± 0.021**
**Thyroxine**	0.138 ± 0.021	0.338 ± 0.073
**Thiamazole**	0.174 ± 0.022	0.449 ± 0.034**
**Growth hormone**	0.128 ± 0.009	0.353 ± 0.055
**Octreotide**	0.166 ± 038	0.312 ± 0.046
**Histamine**	0.208 ± 0.030**	0.300 ± 0.097
**Diphenhydramine**	0.092 ± 0.012†	0.166 ± 0.035‡

Averages and the standard error for 3–4 mice are indicated. *P < 0.01, **P < 0.05 by one-way ANOVA using Dunnett Test for multiple comparisons with saline treatment as a control. ^†^*P* < 0.05, by one-way ANOVA using Newman–Keuls Test for multiple comparisons with gonadotropin-treatment as a positive control. ^‡^*P* < 0.01, by one-way ANOVA using Newman–Keuls Test for multiple comparisons with nandrolone-treatment as a positive control.

### Effect of drugs on the early regeneration of damaged mucosa and the survival rate of mice

**Fig. 2.**
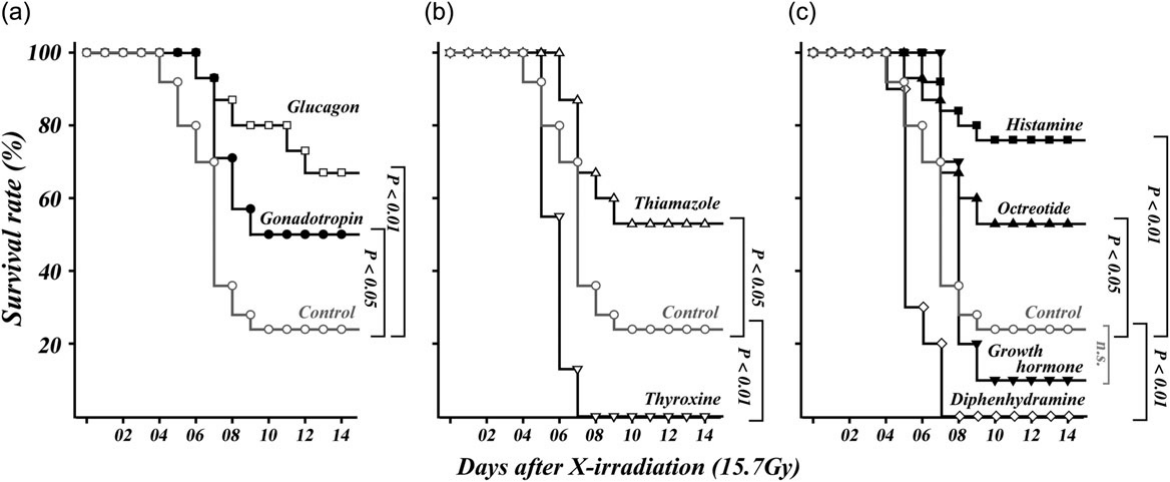
Effect of drugs on the survival curve of mice exposed abdominally to 15.7 Gy of X-rays. Indicated drugs or saline (control, gray) were administered from Day 1 to Day 7 after the irradiation. (**a**) Comparison of glucagon (open square) as positive control, and gonadotropin (closed circle). (**b**) Effect of thyroxin (open downward triangle), or thiamazole (open upward triangle). (**c**) Effect of growth hormone (closed upward triangle), octreotide (closed downward triangle), histamine (closed square) or diphenhydramine (open diamond). *P*-values determined by log-rank test following the Kaplan-Meier method are indicated.

The effect of anabolic steroid (at 0.2–20-fold standard dose) on mucosal epithelial growth [9] suggests that gonadal regulation plays a role in mucosal regeneration in the intestine. Since androgens have anabolic actions, we examined the action of gonadotropin, a stimulator for release of endogenous androgen in male mice. When gonadotropin was administered daily (at the dose corresponding to the maximal daily dose) from day after whole-body irradiation at 15.7 Gy, significant increases were observed in the mucosal *c-myb* mRNA rate on Days 4 and 5 (Table 1), and in the BrdU-positive microcolony rate in the mucosa on Day 4 (Table 2). This means gonadotropin contributes to the proliferation of stem cells on Days 4 and 5, and the expansion of the epithelial area by Day 4. The profiles were different from the action of the anabolic steroid, nandrolone, which significantly activates both indicators on Day 5 (Tables 1 and 2). A similar response of stimulation on Day 5 is also observed after daily injection of a 10-fold dose of glucagon, a gastrointestinal hormone known to stimulate intestinal mucosal growth [6]. Significant survival-enhancing effects on abdominally irradiated mice were observed with both glucagon and gonadotropin (Fig. 2a).

Thyroid hormone was examined since it is also expected to stimulate growth of the intestinal mucosa [19]. Unexpectedly, administration of thyroxin at the standard dose did not produce any difference in the regeneration parameters of the severely damaged mucosa. Only when a large amount (corresponding to a 125-fold daily dose of thyroxin) was injected, were increases in *c-myb* mRNA observed on Days 4 and 5, but these increases were not statistically significant (Table 1). Additionally, microcolony rates on Days 4 and 5 were not elevated (Table 2). In contrast, administration of a standard dose of thiamazole (i.e. an inhibitor of thyroid hormone synthesis) significantly enhanced *c-myb* expression and microcolony generation by Day 5 (Table 1). For abdominally irradiated mice, thiamazole at the standard dose increased the survival rate significantly, but a large amount of thyroxine affected the survival rate negatively (Fig. 2b).

After administration of five times the standard dose of growth hormone, the c-myb RNA rates tended to be increased by Days 4 and 5 (Table 1); microcolony growth appeared to be enhanced at Day 5 (Table 2), and the survival rate slightly decreased (Fig. 2c), but none of these effects were significant. Similarly, octreotide (used as an inhibitor of growth hormone secretion) stimulated the *c-myb* RNA rate observed at Day 4 and 5, but without statistical significance (Table 1), and showed no effects on microcolony growth rate (Table 2). However, high-dose (twice standard dose) octreotide had a significant benefit to the survival of the abdominally irradiated mice (Fig. 2c).

Finally, we examined histamine as an example of a biogenic amine without direct effects on cell growth. The histamine level in the small intestine ( ∼ 20 nmole/g of mucosa) [10] is known to be increased to 50 nmole/g by inflammation due to trypanosomiasis [20]. An increase in the mucosal histamine level is also reported in the case of radiation enteritis [11]. To elevate the histamine level, we injected histamine dichloride at a dose of 40 mg/kg body weight (20% dose of lowest published toxic dose in murine recorded in RTECS), corresponding to 220 nmole/g-body weight. The injection of histamine induced a robust survival-enhancing effect on the abdominally irradiated mice (Fig. 2c), even though partial enhancement of *c-myb* mRNA (Table 1) and significant growth (Table 2) were only observed at Day 4. These results suggest that the physiological level of mucosal histamine may protect epithelial cells. To inhibit the action of the endogenous histamine in the mucosa, we injected diphenhydramine, a typical histamine H1 receptor antagonist [21]. It has been estimated that 10 nmole/ml of diphenhydramine is necessary to suppress the effect of histamine at 50 nmole/ml on ion transport of rabbit ileal mucosa [22]. To suppress the effect of endogenous histamine, a dose of 90 nmole/g-body (30 mg/kg, 20-fold clinical dose) of diphenhydramine hydrochloride was injected. Under the treatment, mucosal microcolony formation was significantly inhibited as observed at Day 4 and 5 (Table 2), although significant stimulation of *c-myb* expression was observed at Day 4 (Table 1). All the abdominally irradiated mice died, probably due to the strong inhibiting action of histamine (Fig. 2c).

## DISCUSSION

Radiation intestinal syndrome is sometimes critical, but there is no standard treatment and only symptomatic therapies have been proposed [23]. In the future, regeneration therapeutics such as mesenchymal stem-cell transplantation [24] shall be established, and further developed by research on epithelial stem cells in the crypts of the small intestinal mucosa [18]. However, the current range of treatments (for which the safety and risk have been evaluated) is limited. From the practical point of view, we sought to identify drugs that could be readily used in a clinic to treat severely damaged intestinal mucosa of the small intestine following a radiation accident. To examine the effects of the drugs, early regeneration of the mucosa was modified by a range of hormones believed to regulate mucosal homeostasis. Ultimately, both expected and unexpected results were obtained during the quantitative and statistical studies of the mucosal regeneration and the survival rate of otherwise lethally irradiated mice.

First, we showed that a clinical dose of gonadotropin significantly enhanced the regeneration of damaged mucosa as well as the survival rate (Tables 1 and 2, Fig. 2a). Since gonadotropin is known to stimulate secretion of endogenous androgen, it is presumed that the damaged mucosa may be regenerated by androgens [9]. Thus, further studies focused on the mechanisms mediated by androgen receptors [25] in the mucosa would be valuable for developing a therapy for intestinal radiation damage. On the other hand, glucagon (used in this study as a positive control, based on previous findings [6]) also stimulates mucosal regeneration by Day 5 (Tables 1 and 2), and also improves the probability of survival when administered at 10-fold standard dose (Fig. 1a). This is consistent with previous reports showing that glucagon-like peptide-2 (GLP-2) cures intestinal mucosal damage at 10–200-fold standard dose [7, 8]. Since the mucosal growth is mediated by phosphatidyl inositol-3 kinase at 50-fold standard dose of GLP-2 [26], several processes independent of the androgen axis may be contributing to the damage repair.

Second, clinical doses of thiamazole, an inhibitor of thyroid hormone synthesis, stimulated mucosal growth, as observed at Day 4 and 5 (Tables 1 and 2), and also showed survival benefits (Fig. 2b). On the other hand, it has been reported that thyroid hormone receptor-alpha signal prevents degradation of the crypts until 48 h after radiation [27]. On this basis, the degradation process (0–48 h after irradiation) and the regeneration process, including mucosal growth (four days after exposure), in the crypts should be investigated separately in the study of intestinal radiation damage. In the case of mucosal growth, thyroid hormone is known to stimulate proliferation of intestinal mucosal cells during development [19] and metamorphosis of *Xenopus laevis* [28]. However, in this study mucosal growth was not accelerated in irradiated mice by thyroxin given at the standard dose. Enhancement of the mucosal *c-myb* mRNA level was observed only after a large dose of thyroxin was used (Table 1), and this was not reflected in proliferation of epithelial cells (Table 2); the survival rate was decreased by its toxicity (Fig. 2b). This suggests that the growth-stimulating mechanism driven by thyroxin did not fully function in the severely damaged mucosa. Conversely, thiamazole given at a dose to inhibit synthesis of endogenous thyroid hormone enhanced mucosal regeneration, as observed at Day 5, as well as the final survival rate (Tables 1 and 2, Fig. 2b). The thiamazole is known as a radical scavenger, and is used to avoid dermatitis by protecting from ultraviolet radiation [29]. Thiamazole not only reduces mucosal oxidative damage [30], but also suppresses inflammation by repression of pro-inflammatory cytokines in colitis when 5 mg/kg of phenyl thiamazole is injected [31]. Therefore, administration of thiamazole may prevent inflammatory aggravation of the mucosa of the small intestine following radiation, and thus enhance the regenerative process.

Third, octreotide, which is known to suppress growth hormone secretion in the pituitary, enhanced survival of the mice (Fig. 2c) when a high dose (2-fold standard dose) was injected. Pretreatment of rats with octreotide is known to protect the intestine against radiation [32] by inhibition of epithelial proliferation [33]. However, a clinical study showed that pretreatment with a long-acting form of octreotide prior to chemotherapy or radiotherapy for anorectal cancer does not alleviate diarrhea [34]. On the other hand, administration of short-acting octreotide is known to resolve the symptom after onset of radiation-induced diarrhea, and the effectiveness has been proven statistically by clinical investigation [35]. In this study, mucosal regeneration parameters were not significantly changed by octreotide (Tables 1 and 2), indicating that neither direct stimulation nor suppression of mucosal growth had occurred. An early study also reported that octreotide changed the levels of inflammatory parameters during radiation enteritis [36]. Therefore, it is speculated that modification of inflammation by octreotide probably contributed to the survival of the mice in this study (Fig. 2c). This is consistent with the result showing that growth hormone, known to be a radioprotector at a high dose [37], did not show any effects on the intestinal mucosa when it was administered after irradiation in this study (Tables 1 and 2, Fig. 2c).

Fourth, histamine was effective in promoting survival (Fig. 2c), although it is difficult to use it as a medical drug owing to its critical action. Histamine protects radiation damage in intestinal mucosa [10] and hematopoietic tissue [38] when it is injected before irradiation. Since none of the four histamine receptors have direct effects on cell growth, it is understood that elevation of the intestinal histamine level enhances the function of the submucosal tissues, which protect the adjacent epithelial cells [10]. Although the regeneration indicators used in this study are focused on early mucosal growth, the indirect action of histamine was probably reflected in the partial stimulation of *c-myb* mRNA level, as observed on Day 4 (Table 1), and in the significant expansion of the mucosal epithelium on Day 4 (Table 2). The necessity for mucosal histamine was also demonstrated by the aggravating effect produced by a high dose of diphenhydramine (Table 2, Fig. 2c). From these results, it is suggested that the increase in mucosal histamine during radiation enteritis [10] might be useful for enhancing regeneration if an appropriate regimen can be established. Research focused on the mucosal environment of the intestinal lumen is now necessary to further investigate the regeneration of the epithelial crypts following severe radiation damage.

## CONCLUSION

In conclusion, these animal studies showed that clinical doses of gonadotropin and thiamazole, and a high dose of octreotide, are effective drugs to ameliorate radiation mucosal damage in the small intestine, similar to previously reported drugs such as a low dose of anabolic steroid [9], a clinical dose of glucagon [6] and high doses of glucagon-like peptide-2 [7, 8]. These drugs may be considered as options in planning the treatment for patients with severe high-dose accidental radiation, and can be used in the experimental animal model to cure intestinal damage. In this study, obvious mucosal regeneration was observed with the activation of the gonadal hormone system. Neither thyroid hormone nor growth hormone showed an advantage in the repair of intestinal mucosa damaged by radiation. On the other hand, the survival-enhancing effect of thiamazole, which is a typical antagonist of the thyroid hormone system, appeared to be caused by reduction of inflammation. The ameliorative effect of octreotide also appeared to be dependent upon the modification of inflammation, rather than the inhibitory effect on growth hormone. Histamine, a typical mediator that acts to modulate the inflammatory response in the mucosa, had strong survival-enhancing effects. Thus, it is speculated that advertent modification of the early mucosal damage process following radiation, focusing on the detailed molecular mechanism, will be necessary to develop a practical therapeutic methodology.

## FUNDING

This study was funded by the Advanced Medical and Radiological Science Division of the Research Promotion Bureau of the Ministry of Education, Culture, Sports, Science and Technology of the Japanese Government.
